# Environmental disinfection with photocatalyst as an adjunctive measure to control transmission of methicillin-resistant *Staphylococcus aureus*: a prospective cohort study in a high-incidence setting

**DOI:** 10.1186/s12879-018-3555-1

**Published:** 2018-12-03

**Authors:** Min Hyung Kim, Seong Gyu Lee, Ki Sook Kim, Yoon Ji Heo, Ji Eun Oh, Su Jin Jeong

**Affiliations:** 1 Department of Internal Medicine, Bundang Jesaeng Hospital, 180-2Seohyeon-ro, Bundang-gu, Seongnam-si, Gyeonggi-do 463-774 South Korea; 2 Department of Laboratory Medicine, Bundang Jesaeng Hospital, 180-2Seohyeon-ro, Bundang-gu, Seongnam-si, Gyeonggi-do 463-774 South Korea; 3 Department of Infection Control Office, Bundang Jesaeng Hospital, 180-2Seohyeon-ro, Bundang-gu, Seongnam-si, Gyeonggi-do 463-774 South Korea; 4 Department of Nursing, Bundang Jesaeng Hospital, 180-2Seohyeon-ro, Bundang-gu, Seongnam-si, Gyeonggi-do 463-774 South Korea; 5 Department of Internal Medicine and AIDS Research Institute, Yonsei University College of Medicine, 50-1 Yonsei-ro, Seodaemun-gu, Seoul, 03722 Republic of Korea

**Keywords:** Photocatalyst, Environmental disinfection, MRSA, Hospital-acquired infection

## Abstract

**Background:**

Environmental disinfection with continuously antimicrobial surfaces could offer superior control of surface bioburden. We sought to decide the efficacy of photocatalyst antimicrobial coating in reducing methicillin-resistant *Staphylococcus aureus* (MRSA) acquisition in high incidence setting.

**Methods:**

We performed prospective cohort study involving patients hospitalized in medical intensive care unit. A titanium dioxide-based photocatalyst was coated on high touch surfaces and walls. Five months of pre-intervention data were compared with five months of post-intervention data. The incidence rates of multidrug-resistant organism acquisition and the rates of hospital-acquired blood stream infection, pneumonia, urinary tract infection, and *Clostridium difficile*–associated diseases were compared using Cox proportional hazards regression analysis.

**Results:**

In total, 621 patients were included. There was significant decrease in MRSA acquisition rate after photocatalyst coating (hazard ratio, 0.37; 95% confidence interval, 0.14–0.99; *p* = 0.04). However, clinical identification of vancomycin-resistant *Enterococcus spp.* and multidrug-resistant *Acinetobacter baumannii* did not decrease significantly. The hazard of contracting hospital-acquired pneumonia during the intervention period compared to baseline period was 0.46 (95% confidence interval, 0.23–0.94; *p* = 0.03).

**Conclusions:**

In conclusion, MRSA rate was significantly reduced after photocatalyst coating. We provide evidence that photocatalyst disinfection can be an adjunctive measure to control MRSA acquisition in high-incidence settings.

**Trial registration:**

ISRCTN Registry (ISRCTN31972004). Registered retrospectively on November 19, 2018.

**Electronic supplementary material:**

The online version of this article (10.1186/s12879-018-3555-1) contains supplementary material, which is available to authorized users.

## Background

Multidrug-resistant organisms (MDROs), including methicillin-resistant *Staphylococcus aureus* (MRSA), vancomycin-resistant *Enterococcus spp.*(VRE), and multidrug-resistant *Acinetobacter baumannii* (MRAB), have increased in prevalence in many acute and long-term care facilities [1]. Controlling hospital-acquired infections (HAIs) associated with these organisms has become a major challenge [2, 3].

As patient-to-patient spread is a major route of MDRO transmission, hand hygiene and isolation are pivotal infection control measures [4]. However, compliance with these measures is low [5]. Hospital environments could be source of outbreaks of resistant organisms [6], and experts suggest indirect transmission via the environment to be as likely as direct person-to-person transmission [7]. Although The Society for Healthcare Epidemiology of America recommends environmental cleaning only with moderate strength [4], enhanced environmental cleaning can reduce MDRO transmission [7–9].

Several technologies have proven antibacterial efficacy and these include hydrogen peroxide vapor [10–14], ultraviolet (UV) light decontamination [15, 16], and copper- and silver-coated surfaces [17–19]. However, their use is limited by high cost or high risk of recontamination.

Among currently recognized alternatives are photocatalytic antimicrobial coating. Photocatalysis mainly uses a semiconductor such as titanium dioxide (TiO_2_) that can absorb UV wavelength < 400 nm and stimulate reactions on its surface producing highly reactive oxygen species (ROS), contributing to biocidal activity. Presently, further improvements such as composite TiO_2_ have enabled activation under visible light, increasing availability in hospital settings [20]. Since this material is stable with respect to self-destruction and responsiveness to light, it is presumed to have durable antimicrobial activity [21]. Furthermore, TiO_2_ has better safety data than pure copper- and silver-coated surfaces, especially when exposed in low doses, such as in environmental coating [22–24].

Although many in vitro studies have presented positive results of photocatalytic antimicrobial action with titania, only little work in real life application has been reported. These works raise numerous issues about applying this material in hospital environment. Inappropriately incorporated binder, which acts as a glue to adhere TiO_2_ to the surface, could prevent the action of photocatalyst [25]. Even if properly manufactured, slow action time of photocatalyst makes it act as a supplemental measure to the conventional decontamination procedures at best [26]. Furthermore, others argue that its effect is not significant on already thoroughly cleaned surfaces [27].

Recognizing the significance of these issues, we decided to use metal ion-doped nanoparticle TiO_2_, which is firmly attached to the surface, as adjunctive measure of environmental decontamination. Additionally, we recognized high incidence rates of MRSA at this institute (3-year average incidence from 2014 to 2016 was 7.7/1000 patient-days). Taken together, we sought to evaluate the usefulness of a photocatalyst to reduce the risk of MRSA transmission, by comparing MRSA acquisition rate and nosocomial infection rate before and after photocatalyst coating.

## Methods

### Setting

The study was performed at a 630-bed secondary care teaching hospital in Gyeonggi-do, South Korea. This institution has two adult intensive care units (ICUs), medical and surgical, with 14 and 15 beds each, and has an average of 600–650 admissions annually. Medical ICU is a single room, composed of three sections that are interconnected. We only included the medical ICU in this study.

### Study design

We performed a prospective cohort study involving patients hospitalized in the medical ICU between September 2016 and June 2017. The study was divided into 5-month baseline and post-intervention periods: baseline period, September 2016 to January 2017; intervention period, February 2017 to June 2017. Photocatalyst was uniformly coated on high tough surfaces and walls at the end of January 2017. All patients admitted to the medical ICU during the study period were included with the exception of patients aged < 18 years and patients hospitalized less than 72 h. Only first episode of ICU admission was included in the analysis, with the exception of a second episode that was ≥3 months from the previous episode.

Routine infection control procedures were maintained throughout the study period, including hand hygiene monitored by infection control nurses (142 to 176 observations per month), isolation of patients with known transmissible disease, and conforming to ICU bundle recommended by The Centers for Disease Control and Prevention guidelines [28].

Surface areas, including patient body surfaces, were cleaned throughout the study period. All ICU patients were washed with chlorhexidine-impregnated cloth every 3 days. High-touch surfaces such as upper and lower benches, computer keyboards and mouse, telephones in the nursing station, and mobile equipment such as blood pressure monitoring machines, items in blood collection trolleys, and bed rails were cleaned with chlorhexidine-impregnated cloth. Floors of the rooms of non-isolated patients were cleaned daily with neutral detergent, while sodium hypochlorite (1000 ppm) was used for cleaning areas with known VRE, *Clostridium-difficile*–associated disease (CDAD) patients. At this institute, annual general deep cleaning of ICU, which comprises thorough cleaning with neutral detergent followed by waxing after moving patients to other wards, had been taking place, usually at the end of the year or in January of the next year.

The primary outcome of the study was incident MRSA acquisition rate. Nursing staff obtained swabs from patients’ nares up to 48 h after ICU admission and on discharge from ICU. A new MRSA acquisition was defined as a patient not positive for MRSA on admission who subsequently became positive following screening culture after admission. The term “acquisition” was considered synonymous with “colonization” or “infection” obtained after negative culture on ICU admission. The nasal swabs were plated onto chromogenic media (bioMérieux, Marcy l’Etoile, France) for *S. aureus* detection. Subsequently, *S. aureus* was subcultured on Mueller–Hinton Agar plus cefoxitin for investigating cefoxitin resistance.

Secondary outcome measures included VRE and MRAB isolates from clinical sites, CDAD and HAIs (bloodstream infection, pneumonia, and urinary tract infection) as defined by The Centers for Disease Control and Prevention [29]. Microorganisms and their antibiotic sensitivities were identified using Vitek2 (bioMérieux, Marcy l’Etoile, France). MRAB was defined as resistance to more than three classes of antibiotics. CDAD was diagnosed by real-time polymerase chain reaction for *C. difficile* toxin A and B gene or by stool culture for patients having diarrhea.

Specimens for environmental surveillance cultures were collected by infection control nurses to assess extent of contamination or disinfection. Thirty high-touch surfaces including bedside rails, tabletops, nursing trolley tops, door handles, tap handles, and computer keyboards and mouse were sampled. Surveillance cultures were performed bimonthly, 3 times each during the baseline and post-intervention periods. Polywipe sponge swabs (Medical Wire & Equipment, Corsham, UK) were used, and imbedded in thioglycollate medium (Becton Dickinson, SA, France) and cultured for detection of microorganisms. The results were shown as percentage of positive bacterial cultures against entire cultures performed.

### Intervention

We used a nanoparticle TiO_2_ doped with transition metal ion (iron included), which can respond to visible light (NexChem, Chungcheongbuk-do, South Korea). The area was vacated for 24 h after which the photocatalyst was applied and dried. The unit was thoroughly cleansed before photocatalyst application. An electrostatic spray was used to generate atomized droplets that were deposited uniformly on various surfaces. It was then dried to form a tough, adherent monolithic film on the coated surface. All plastic and metal surfaces such as upper and lower benches, bedside rails, tabletops, nursing trolley tops, door handles, tap handles, telephones in the nursing station, blood pressure monitoring machines and walls were sprayed. The procedure commenced on January 26, 2017, and ended on January 27, 2017.

### Data collection

We collected baseline clinical data, including patient age and sex, length of ICU stay, length of hospital stay, pre-existing chronic comorbidities (diabetes, chronic heart failure, chronic liver disease, chronic renal disease, chronic pulmonary disease), sequential organ failure assessment score, admission history within 3 months prior to ICU admission, previous invasive procedure (central line insertion, intubation, continuous renal replacement therapy, surgery under general anesthesia), and length of antibiotics treatment.

Hospital-acquired pneumonia, BSI, and UTI were defined based on The Centers for Disease Control and Prevention criteria. The absolute numbers of infection cases was divided by total patient-days and described as events/1000 patient-days.

### Statistical analysis

Patients who stayed across baseline and post-intervention periods in the ICU were excluded from analysis. Baseline characteristics were compared using Mann–Whitney U test or independent samples *t*-test for continuous variables, and χ^2^ test or Fisher’s exact test for categorical variables. Continuous variables were expressed as means, or medians (interquartile ranges) and categorical variables as numbers with percentages for the description of baseline characteristics.

The intervention effect was measured in terms of hazard and incidence rate ratios. The relative hazard of MDROs acquisition was calculated using a Cox proportional hazards model, adjusted for clinically relevant confounders in addition to variables that showed statistical differences in univariate analysis. The proportional hazards assumption was tested by including an interaction term between variables. We excluded variables that did not fit the assumption of proportionality. Patients were categorized into two groups as baseline and intervention period. Survival curves were created using the Kaplan–Meier method and compared using the log-rank test. Variables were considered significant at *p* < 0.05, and the results were presented as hazard ratios with 95% confidence intervals (CIs). Subgroup analysis stratified by length of ICU stay was conducted to identify either consistency of or differences in the magnitude of intervention effect.

Statistical analyses were performed using R software version 3.4.2 (R Development Core Team 2016; http://www.r-project.org/).

## Results

### Patient characteristics

**Fig. 1:**
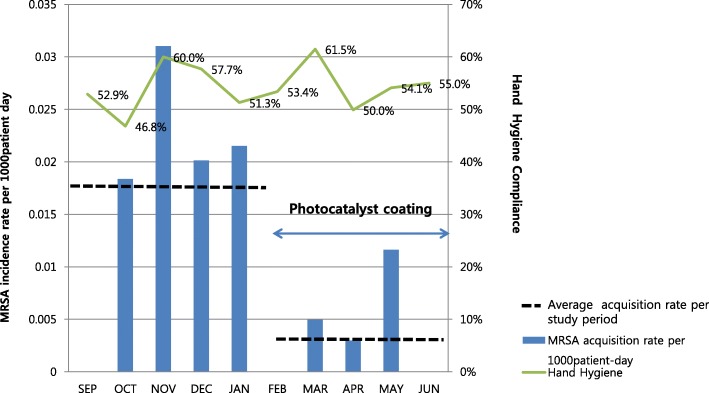
Methicillin-resistant *Staphylococcus aureus* acquisition rate during the baseline and intervention periods, and compliance of hand hygiene

In total, 858 patients were admitted to the ICU during the study period. Among them, 233 were excluded due to young age or short length of hospital stay and 4 due to staying across baseline and post-intervention periods. Finally, 341 and 280 patients during baseline and intervention periods, respectively, were enrolled. Therefore, data from 621 patients were analyzed. Mean age of enrolled patients was 67.56 years and 59.5% were men. Mean length of ICU stay was 4.87 days: baseline period, 4.30 days; intervention period, 5.40 days. Mean length of hospital stay was 23.50 days: baseline period, 21.07 days; intervention period, 26.46 days. Overall mortality was 13.7%. There was no significant difference in underlying diseases between the groups except for number of patients having chronic renal disease, which was slightly higher in the intervention group (18.1% vs. 25.5%, *p* = 0.03). Previous admission history, length of antibiotics treatment, admission route, and sequential organ failure assessment score were not statistically different. Invasive procedures were conducted equally in both groups. The proportion of MRSA colonizers who acquired MRSA before ICU admission was same between the groups (Table 1). The compliance of hand hygiene of physicians remained low across study periods (Fig. 1).

**Table 1:** Baseline characteristics of the study population

Variables	Total	Baseline	Intervention	*p*-value
Patients, n	621	341	280	NA
Male gender, n (%)	370 (59.5)	199 (58.4)	171 (61.1)	0.55
Age (years)	67.56	67.13	68.08	0.43
Total patient days at risk	3171	1613	1558	0.24
Mean length of hospital stay	23.50	21.07	26.46	0.12
Mean length of ICU stay	4.87	4.30	5.40	0.11
Overall mortality, n (%)	85 (13.7)	48 (14.0)	37 (13.7)	0.91
Previous admission Hx. within 3 months, n (%)	139 (22.4)	66 (19.4)	73 (26.1)	0.06
Comorbidities				
Cardiovascular disease, n (%)	453 (72.9)	247 (72.2)	212 (75.2)	0.41
Diabetes, n (%)	195 (31.4)	111 (32.5)	84 (29.8)	0.49
Cerebro vascular accident, n (%)	264 (42.5)	146 (42.7)	118 (42.0)	0.87
Solid organ malignancy, n (%)	75 (12.1)	43 (12.6)	32 (11.3)	0.71
Hemaologic malignancy, n (%)	4 (0.6)	3(0.9)	1 (0.4)	0.63
Trauma Hx., n (%)	20 (3.2)	14 (4.1)	6 (2.1)	0.18
Chronic renal disease, n (%)	133 (21.4)	61 (18.1)	72 (25.5)	0.03
Chronic liver disease, n (%)	70 (11.3)	36 (10.5)	34 (12.1)	0.61
Chronic lung disease, n (%)	82 (13.2)	44 (12.9)	38 (13.5)	0.91
Connective tissue disease, n (%)	13 (2.1)	6 (1.8)	7 (2.5)	0.58
SOFA score	4 (2–7)	4 (2–7)	4 (2–6)	0.38
Invasive procedure				
Central line catheter insertion, n (%)	273 (44.0)	155 (45.3)	118 (41.8)	0.42
Intubation, n (%)	167 (26.9)	98 (28.7)	69 (24.5)	0.28
CRRTx.,n (%)	38 (6.1)	22 (6.4)	16 (5.7)	0.74
Operation history, n (%)	92 (14.8)	51 (15.0)	41 (14.6)	1.00
Median duration of antibiotics treatment	6 (0–15)	7 (0–15)	6 (0–16)	0.59
Vancomycin use, n (%)	70 (11.3)	36 (10.6)	34 (12.1)	0.61
MRSA acquisition prior to ICU admission	57 (9.2)	36 (10.5)	21 (7.5)	0.21
ICU bed occupancy, %	82.0	83.7	81.5	0.22
Hand hygiene compliance of all HCW	72.1	71.6	72.7	0.62
Hand hygiene compliance of physicians	54.2	53.8	54.6	0.48
Hand hygiene compliance of nurses	89.9	89.4	90.5	0.64

Data are expressed as the mea / median (Q1-Q3) or N (%)

*Abbreviation: NA* not applicable, *ICU* intensive care unit, *SOFA* sequential organ failure assessment, *CRRTx*., continuous renal replacement therapy, *HCW* health care workers

### Acquisition of MRSA

**Fig. 2:**
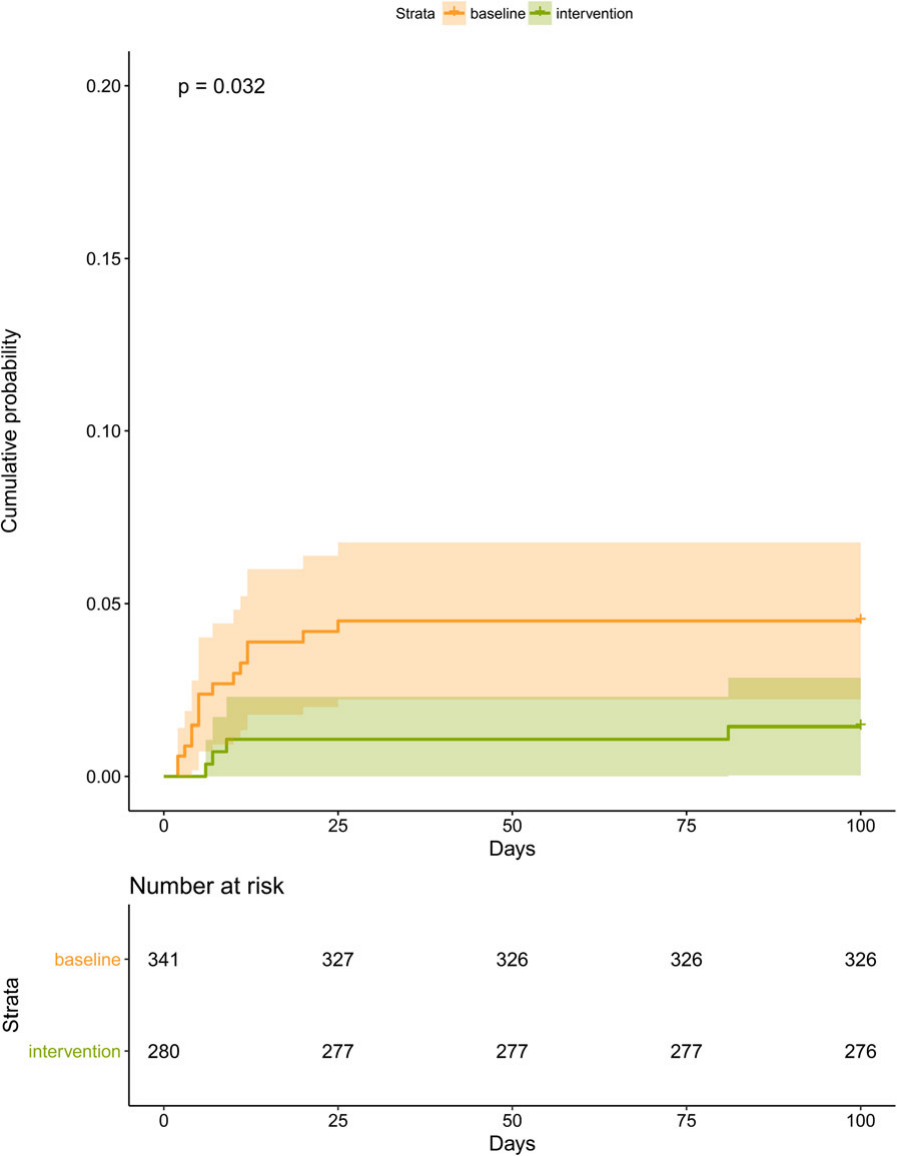
Kaplan–Meier estimates of time to methicillin-resistant *Staphylococcus aureus* acquisition. The cumulative probability of methicillin-resistant *S. aureus* acquisition is shown for patients in the baseline vs. intervention period. The relative hazard of acquiring methicillin-resistant *S. aureus* in the intervention vs. baseline period was 0.37(95% confidence interval, 0.14–0.99; *p* = 0.04).

During baseline period, 15 newly acquired MRSA cases were detected compared with 4 during the intervention period. Incidence rate reduced from 9.3/1000 patient-days before intervention to 2.57/1000 patient-days after intervention. Cox proportional hazards survival regression analysis showed the risk of acquiring MRSA was significantly lower in the intervention period than in the baseline period (*p* = 0.03) (Fig. 2). After adjusting for sequential organ failure assessment score, renal function, and length of ICU stay, the hazard of acquiring MRSA during the intervention vs. baseline period was 0.37 (95% CI, 0.14–0.99; *p* = 0.04). This effect was greater among patients with longer length of stay. Among patients who stayed longer than 7 days in ICU, the relative hazard was 0.26 (95% CI, 0.07–0.96; *p* = 0.04). Figure 2 shows MRSA acquisition rates stratified by date of admission between baseline and intervention periods. MRSA acquisition rate was significantly reduced after photocatalyst application and remained low throughout the intervention period.

The clinical identification of VRE remained low across study periods and had not been significantly reduced after intervention (0.62 vs. 1.28/1000 patient-days, *p* = 0.54). Incidence rate of MRAB also did not decrease significantly (3.09 vs. 3.20/1000 patient-days, *p* = 0.76) (Table 2).

**Table 2:** Comparison of MDRO acquisition rate and hospital acquired infection rates by study period

Variable	Baseline	Intervention	IRR	95% CI	*p*-value	HR^a^	95% CI	*p*-value
MRSA acquisition, n (%)	15 (4.4)	4 (1.4)			**0.01**			
MRSA acquisition rate per 1000 patient-days	9.30	2.57	0.26	0.06–0.81		0.37	0.14–0.99	**0.04**
VRE acquisition, n (%)	1 (0.9)	2 (1.1)			0.54			
VRE acquisition rate per 1000 patient-days	0.62	1.28	2.07	0.19–22.84				
MRAB acquisition, n (%)	5 (6.4)	5 (8.5)			0.76			
MRAB acquisition rate per 1000 patient-days	3.09	3.20	1.03	0.30–3.57				
Blood stream infection, n (%)	6 (1.8)	10 (3.5)			0.28			
Blood stream infection rate per 1000 patient-days	3.71	6.41	1.72	0.63–4.75				
Pneumonia, n (%)	26 (7.6)	12 (3.2)			**0.03**			
Pneumonia rate per 1000 patient-days	16.12	7.70	0.48	0.24–0.95		0.47	0.23–0.94	**0.03**
Urinary tract infection, n (%)	9 (2.6)	5 (1.8)			0.32			
Urinary tract infection rate per 1000 patient-days	5.58	3.21	0.57	0.19–1.71				
CDAD, n (%)	2 (0.6)	1 (0.4)			0.58			
CDAD rate per 1000 patient-days	1.23	0.64	0.52	0.05–5.70				

The incidence rate ratio was obtained by dividing the incidence rate in intervention period by the incidence rate in baseline period

Results having stastical significance was presented in boldface

*Abbreviation: IRR* incidence rate ratio, *CI* confidence interval, *HR* hazard ratio, *MRSA* methicilline resistant *S.aureus*, *VRE* vancomycin resistant *Enterococcus spp*., *MRAB* multidrug resistant *A.baumannii*, *CDAD Clostridium difficile* associated diarrhea

^a^Hazard ratio was calculated using a multivariate Cox proportional hazards model, adjusted for length of ICU stay, SOFA score and having or not chronic renal diseases

### HAI rate

There was significant reduction in incidence rate of hospital-acquired pneumonia (baseline vs. intervention period: 16.12/1000 vs. 7.70/1000 patient-days; *p* = 0.03). The hazard of acquiring pneumonia during the intervention vs. baseline period was 0.47 (95% CI, 0.23–0.94; *p* = 0.03). Pathogens were identified in 76.3% of cases. The most common pathogen was Gram-negative bacilli (*n* = 22, 57.9%). MRSA was isolated in 13.16% (*n* = 5) of cases. However, no statistically significant reduction in BSI, UTI, or CDAD was observed. The incidence of BSI was 3.71/1000 vs. 6.41/1000 patient-days in baseline and intervention periods (*p* = 0.28). Incidence rate of UTI was 5.58/1000 vs. 3.21/1000 patient-days in baseline and intervention periods (*p* = 0.32). The incidence rate of CDAD was 1.23/1000 vs. 0.64/1000 patient-days in baseline and intervention periods (*p* = 0.52) (Table 2).

### Environmental disinfection

Statistically significant decrease of microorganisms on high-touch surfaces was observed (27/90 [30%] vs. 10/90 [11%], baseline and intervention period respectively; *p* = 0.01). Immediately after the intervention, significantly lower number of microorganisms was isolated from the surfaces. Thereafter, lower levels of organisms in the intervention period than in the baseline period were cultured from the surfaces; however, it was not statistically significant. The most common organism isolated was coagulase-negative *Staphylococcus spp.* (*n* = 33, 89.2%). The rest were *Bacillus spp.* Details of environmental culture results are provided in supplemental table (see Additional file 1: Table S1).

## Discussion

Photocatalysts are investigative materials for its efficacy in controlling MDRO in hospital environments. Our study suggests that photocatalysts are effective in controlling MRSA acquisition. However, it has limited efficacy on MRAB. Proper evaluation of its effect on VRE was not possible due to paucity of newly acquired cases.

In Asia Pacific region, MRSA remains a threat, accounting for > 50% of *S. aureus* infections, and MRSA BSI was reported to be 14.4/10,000 patient-days in 2017 [30]. In such high-prevalence settings, there has been growing interest in the horizontal approach, such as chlorhexidine bathing, hand hygiene, and environmental cleaning instead of isolation. Among these, hand hygiene itself could be cost-effective way of prevention; however, it is often difficult to sustain, especially when monitoring staff is scarce and turnover rate of housekeeping staff is high. One of the reasons for high MRSA prevalence at this institute could be that hand hygiene was practiced at moderate to below average level. Only after temporary closure of wards had been instituted and full decontamination of the departments using photocatalyst was performed did the MRSA acquisition rate reduce.

Stiefel et al. suggested that healthcare workers’ hands were equally contaminated from contact with commonly touched environmental surfaces as from direct contact with colonized patients [31]. Moreover, MRSA can exist on surfaces for as long as 360 days [32, 33], increasing its probability to be transmitted. Our result is promising in that photocatalyst enhanced the effect in combination with regular infection control practices that resulted in lower MRSA acquisition. This result is consistent with previous in vitro evidence, which was conducted in aqueous solution demonstrating positive antimicrobial effect of iron-doped TiO_2_ on *S. aureus* [34].

It is meaningful to note that MRSA acquisition rate remained low throughout intervention period. Although pure TiO_2_ is considered indefinitely active, very few real-time studies concerning durability of TiO_2_ coating have been reported. Instead, laboratory-stimulated endurance test is performed as a substitute for real-time study [35]. The TiO_2_ coating we used was checked for its durability with weather-O-meter test (Testing system for weathering performances CI5000, ATLAS). Nevertheless, existing real-time studies of composite TiO_2_ coating provide positive results in terms of durability. Silver-doped TiO_2_ produced in Japan under the name of MVX reported to remain active after 2 years of lab test, according to data provided by the manufacturer [27]. Furthermore, decontamination with zinc containing TiO_2_ in a long-term care facility reduced HAIs even after 17 months [36]. Their result was consistent with our result on MRSA. The effect was greater among patients who stayed longer than 7 days in ICU. It is possible that 72% reduction of MRSA acquisition was attributed to this durable antimicrobial activity of photocatalyst. Notwithstanding other environmental cleaning methods such as hydrogen peroxide, ultraviolet light, and copper-coated surfaces that proved effective in controlling MRSA [15, 37, 38], photocatalysts still have the advantage of easy application and durability. However, there is concern that the effect is less prominent in stringently cleaned settings. A study from the Netherlands conducted in an ICU, where strict cleaning is a priority, found no microbiological benefit of photocatalyst [27]. However, that study was conducted over a short period and further study is needed to verify its efficacy in low-incidence settings.

We found no beneficial effect of photocatalyst on MRAB. The photocatalysts we used lacked well-established data of its antibiotic efficacy on MRAB. Although visible light-activated composite TiO_2_ is generally expected to have enhanced antimicrobial activity compared to pure TiO_2_, only a limited number of organisms had been tested in laboratory settings. Most of the studies used *E.coli* as representative of gram-negative organism, and *S.auerus* as representative of gram-positive organism [39]. It is not reliable to conclude that these materials are capable of inactivating various kinds of microorganism beyond the scope of organisms tested in laboratory settings. MRAB has different structural properties in terms of outer membrane and number of porins, compared to *E.coli* [40]*.* Since affinity of ROS to outer membrane determines its antibacterial property, different organisms can have different sensitivity to photocatalyst [41]. Although we did not conduct active surveillance and only counted incidental isolates of MRAB, this questionable characteristic can partly explain why MRAB incidence did not decrease. Notwithstanding, we admit that the number of cultured MRAB was small. Further study using larger number of patients for longer period of time is warranted.

We did not observe any trend on clinical endpoints of VRE due to low rate of these endpoints. Our study was underpowered to detect clinically significant differences in these endpoints because average length of ICU stay of our study population was relatively short.

Reduced incidence of hospital-acquired pneumonia was noted, while BSI and UTI were not significantly reduced. Slightly higher percentage of cases of pneumonia was caused by MRSA than by other infections. Only two cases of BSI were caused by MRSA, while 5/29 cases of culture-proven pneumonia were thought to be related to MRSA. We cautiously concluded that reduced MRSA acquisition partly explained decreased incidence of hospital-acquired pneumonia. Additionally, considering more than 50% of isolated organisms were gram-negative bacteria, undetected benefits of photocatalyst may have led to positive result. However, this hypothesis is not robust due to the lack of data supporting it, and study period was relatively short for reduced colonization translated into reduced infection. Moreover, incidence of pneumonia could have been affected by various unquantifiable factors, such as skill level of medical personnel.

It is most likely that reduced MRSA acquisition resulted from reduced burden of MRSA on the environmental surfaces. However, our environmental culture surveillance failed to support the hypothesis. No MRSA was isolated on the surface culture. We did not use dipslide, which was a highly recommend tool to collect MRSA from the environment, because this equipment was not widely available in Korea. This could have underestimated the burden of MRSA in the ICU environment. Nonetheless, significant decrease of coagulase-negative *Staphylococcus spp.* on environmental surfaces can be considered as surrogate marker of environmental decontamination.

We acknowledge several limitations in our study. First, general deep cleaning which was conducted generally at the end of the year could act as confounding. We cannot exclude that previous deep cleaning reduce MRSA acquisition, and photocatalyst coating may serve as a mean to maintain low level of environmental contamination. Second, active surveillance was conducted only during ICU stay, underestimating incidence rate of MRSA from patients who were discharged ICU within 48 h. Third, incidence rate of organisms on which active surveillance had not been conducted could have been under- or overestimated. Finally, efforts to enhance hand hygiene and following effect of this strategy had not been performed. Further study comparing the efficacy of photocatalyst coating with that of enhanced hand hygiene is warranted.

## Conclusions

In conclusion, this study is the first prospective cohort study to evaluate the efficacy of photocatalyst in a practical setting and to provide evidence that photocatalyst disinfection can be an adjunctive measure to control MRSA acquisition in high-incidence settings.

## Additional file

Additional file 1: **Table S1.** Number of positive isolates of environmental culture according to identified organism and surfaces. (DOCX 19 kb)

## Abbreviations

BSI: Bloodstream infection
CDAD: *Clostridium-difficile*–associated disease
CIs: Confidence intervals
HAI: Hospital-acquired infection
ICU: Intensive care unit
MDROs: Multidrug-resistant organisms
MRAB: Multidrug-resistant *Acinetobacter baumannii*
MRSA: Methicillin-resistant *Staphylococcus aureus*
ROS: Reactive oxygen species
TiO_2_: Titanium dioxide
UTI: Urinary tract infection
UV: Ultraviolet
VRE: Vancomycin-resistant *Enterococcus spp*
