# Supplementary material for: Environmental disinfection with photocatalyst as an adjunctive measure to control transmission of methicillin-resistant Staphylococcus aureus: a prospective cohort study in a high-incidence setting
Source: BMC Infect Dis. 2018 Dec 3;18:610. doi: 10.1186/s12879-018-3555-1 (PMC6276245; doi:10.1186/s12879-018-3555-1)
Supplement: Supplementary file 1 — Table S1. Number of positive isolates of environmental culture according to identified organism and surfaces. (DOCX 19 kb) [file 12879_2018_3555_MOESM1_ESM.docx]

**Supplementary table 1**. Number of positive isolates of environmental culture according to identified organism and surfaces

| Name of organism | Surfaces | Number of positive isolates | | p-value |
| --- | --- | --- | --- | --- |
|  |  | Baseline period | Intervention period |  |
| **Coagulase-negative *Staphylococcus spp****.* | Bedside rails | 6 | 2 |  |
|  | Tabletops | 5 | 1 |  |
|  | Nursing trolley tops | 7 | 1 |  |
|  | Door handles | 1 | 1 |  |
|  | Faucet handler | 1 | 1 |  |
|  | Computer keyboards | 3 | 2 |  |
|  | Total (ratio^a^) | 23 (23/90, 0.26) | 8 (8/90, 0.09) | 0.00 |
|  |  |  |  |  |
| ***Bacillus spp.*** | Bedside rails | 2 | 1 |  |
|  | Tabletops | 0 | 0 |  |
|  | Nursing trolley tops | 0 | 0 |  |
|  | Door handles | 1 | 0 |  |
|  | Faucet handler | 0 | 0 |  |
|  | Computer keyboards | 1 | 1 |  |
|  | Total (ratio^a^) | 4 (4/90, 0.04) | 2 (2/90, 0.02) | 0.41 |
|  |  |  |  |  |
| **Total (ratio**^a^**)** |  | 27 (27/90, 0.30) | 10 (10/90, 0.11) | 0.01 |

^a^ Denominators indicate entire environmental culture collected
